# The Removal of Time–Concentration Data Points from Progress Curves Improves the Determination of *K_m_*: The Example of Paraoxonase 1

**DOI:** 10.3390/molecules27041306

**Published:** 2022-02-15

**Authors:** Boštjan Petrič, Marko Goličnik, Aljoša Bavec

**Affiliations:** Institute of Biochemistry and Molecular Genetics, Faculty of Medicine, University of Ljubljana, 1000 Ljubljana, Slovenia; bostjan.petric@mf.uni-lj.si (B.P.); marko.golicnik@mf.uni-lj.si (M.G.)

**Keywords:** paraoxonase 1, dihydrocoumarin, progress curves, integrated Michaelis–Menten equation, Lambert W, lactonase activity

## Abstract

Several approaches for determining an enzyme’s kinetic parameter *K_m_* (Michaelis constant) from progress curves have been developed in recent decades. In the present article, we compare different approaches on a set of experimental measurements of lactonase activity of paraoxonase 1 (PON1): (1) a differential-equation-based Michaelis–Menten (MM) reaction model in the program Dynafit; (2) an integrated MM rate equation, based on an approximation of the Lambert W function, in the program GraphPad Prism; (3) various techniques based on initial rates; and (4) the novel program “iFIT”, based on a method that removes data points outside the area of maximum curvature from the progress curve, before analysis with the integrated MM rate equation. We concluded that the integrated MM rate equation alone does not determine kinetic parameters precisely enough; however, when coupled with a method that removes data points (e.g., iFIT), it is highly precise. The results of iFIT are comparable to the results of Dynafit and outperform those of the approach with initial rates or with fitting the entire progress curve in GraphPad Prism; however, iFIT is simpler to use and does not require inputting a reaction mechanism. Removing unnecessary points from progress curves and focusing on the area around the maximum curvature is highly advised for all researchers determining *K_m_* values from progress curves.

## 1. Introduction

Ever since Leonor Michaelis and Maud Menten first published the equation that bears their name in 1913, the Michaelis constant *K_m_* has been the main kinetic parameter used to quantify the affinity of a given enzyme for its substrate. Traditionally, *K_m_* was determined by measuring the initial rate of the enzymatic reaction at several different substrate concentrations and then plotting the initial rates and substrate concentrations onto one of the linearized transformations of the Michaelis–Menten (MM) equation. These include the linear Lineweaver-Burk, Eadie-Hofstee, and Hanes-Woolf plots, each of which has its own (dis)advantages in terms of precision. With the aid of nonlinear regression methods and computers, it later also became possible to fit an MM model equation directly onto the initial rates without prior linearization of the data. With this, all points are given equal weight during fitting. However, all these approaches share a downside, namely, that only one (initial) rate–concentration data point is available from an individual enzymatic reaction performed in the laboratory.

With further developments in computational methods, it became possible to fit model functions onto entire progress curves, thus rendering MM plots and their derivatives, as well as the measurement of initial rates, unnecessary. There are several advantages to using entire progress curves compared to initial rates: (1) More time–concentration data points are used from each enzymatic reaction, which improves the precision of the final fit; (2) fewer enzymatic reactions need to be performed in total, which reduces the consumption of the enzyme and substrate; (3) the need to start measuring the reaction immediately after it begins is smaller; and (4) the exact amount of substrate present can be calculated from the final plateau of the progress curve itself (for irreversible reactions).

Several computer programs, which rely on several different numerical approaches, allow fitting model functions onto progress curves. One approach is to extract the substrate concentration and velocity for each point on the progress curve and then plot these points onto an MM diagram or its derivatives [1]. However, linearized versions of the MM diagram have the previously noted deficiencies. Another approach is to use a version of the integrated MM equation, which expresses time as a function of product concentration, as a model function. The problem with this approach is that when the progress curve approaches the plateau, the model function performs poorly. The reason for this is that it models time as a function of product concentration, with one of the terms being ln (1 − [P]/[S]_0_); when the progress curve reaches its plateau, this term cannot be calculated anymore [2].

In recent years, an improved integrated version of the MM function has been published, based on the Lambert W function, which expresses product concentration with respect to time [3]. Unfortunately, most general-purpose software packages for data analysis do not include the Lambert W function. To remedy this, Goličnik [4] published a numeric approximation of the above equation that can be used for progress curve fitting in software packages such as GraphPad Prism (referred to henceforth as Prism). Another approach is to fit numerically evaluated theoretical curves based on a system of differential equations onto progress curves; this requires special computer programs such as Dynafit [5] or Enzo [6].

**Figure A1:**
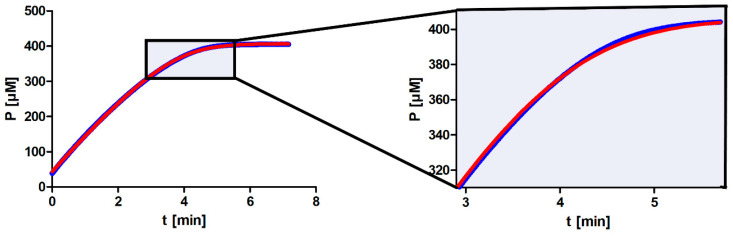
The integrated Michaelis–Menten equation being fitted in Prism to the same progress curve (blue line) that is shown in Figure 3. The model function (red line) appears to be a good fit for the progress curve. However, once we zoom into the area of maximum curvature, it is clear that the fit is no longer as good, which results in an erroneous *K_m_* value.

One persistent problem when working with entire progress curves is deciding which points on the curve should be used to fit the model function. The advantage of model functions such as the integrated MM equation is that they treat all the points on the progress curves the same; however, this can also be a disadvantage. A program can produce a solution that fits perfectly onto the plateau but at the expense of not fitting well onto the area of maximum curvature, which contains the most information about *K_m_* [7]. Such *K_m_* estimates can be considerably far from the true values; see also Figure A1 in Appendix A. A similar problem occurs when *K_m_* is small compared to [S]_0_, as the initial part of the progress curve is almost straight, and any information about *K_m_* can be lost in the noise.

While it is possible to manually trim progress curves and remove points from the initial and final parts of the curve based on intuition or by creating ad hoc rules (e.g., removing all points recorded after reaching the plateau), such approaches are insufficient for obtaining high-quality and reproducible results. An ideal method for data point selection should be based on sound mathematical principles, applicable to different types of progress curves, and capable of producing results as close as possible to real *K_m_* values. 

Such a method has been proposed by Stroberg and Schnell [7]. They proposed an equation that determines the area of maximum curvature of a progress curve from the input values of *K_m_* and *V_max_*. Based on their equation, we have developed an iterative method that determines the area of maximum curvature based on estimates of *K_m_* and *V_max_* and then proceeds to calculate *K_m_* and *V_max_* again from the points within this area. This process continues until the calculated area of maximum curvature remains constant [7]. To calculate *K_m_* and *V_max_* with this iterative method, we selected the integrated MM equation (based on the Lambert W function) and developed a short script in Python that calculates the area of maximum curvature from a progress curve and the resulting *K_m_* and *V_max_* values from this area. The working name of this script is hereafter referred to as iFIT; it is accessible at http://i-fit.si/ (last accessed 29 December 2021).

Several articles have compared different methods for progress curve analysis and have demonstrated their advantages and limitations [8,9,10]. The present article has two major advantages compared to most of these studies: (1) to the best of our knowledge, this is the first study to investigate a method for selecting a sufficient data point area around the time course curvature, and (2) in our study, the different methods have been tested on real experimental data. Most comparisons of methods for progress curve analysis are performed on simulated data; the advantage of such an approach is that the exact *K_m_* values and other kinetic parameters are known [8]. However, although noise is also simulated in such analyses, it is unlikely that this simulated approach fully encapsulates the variety of noise that can occur in an experimental setting. Hence, analysis of simulated progress curves can lead to overly optimistic appraisals of the software used.

This study was based on the enzyme paraoxonase 1 (PON1), which naturally occurs in mammalian blood. PON1 plays an antioxidative role in the bloodstream, presumably via the breakdown of oxidized lipids. PON1 has also been associated with the slower progression of several medical conditions as well as with delayed aging in general and is thus very promising for therapeutic and diagnostic applications [11,12,13]. Since PON1 is naturally associated with high-density lipoproteins and is very difficult to prepare in a native form in cell culture, the use of soluble recombinant versions of PON1 (rePON1) is more convenient for investigating the enzymatic properties of PON1 [14]. According to the literature, the kinetics of recombinant PON1 are especially similar to those of human serum PON1 for arylesterase and organophosphate substrates [14] but somewhat less so to those of lactone substrates [15,16]. Another advantage of rePON1 is its high stability, as reported by the researchers who developed it; G2E6 is not prone to aggregation and could easily be crystallized for structure determination [17].

## 2. Results

### 2.1. The Nonenzymatic Constant for Dihydrocoumarin

Nonenzymatic hydrolysis is a considerable issue when calculating enzymatic parameters from both progress curves and initial rates. Since the spontaneous hydrolysis of dihydrocoumarin (DHC) is a pseudo-first-order reaction, it contributes the most to the total rate of the reaction at a high substrate concentration (at the beginning of the reaction) but contributes the least at a low substrate concentration (in the final part of the reaction).

The nonenzymatic first-order rate constant k_NE_ was derived from the 10 reactions performed in the absence of the enzyme. The rate constant was calculated according to Equation (1):

(1) $$\left[P\right]={\left[S\right]}_{0}\left(1-e^{-k_{\mathrm{NE}}\cdot t}\right)$$

Since the constant is independent of substrate concentration, the 10 calculated k_NE_ values were then averaged, resulting in an k_NE_ value of (0.084 ± 0.003) min^−1^, i.e., t_1/2_ = (8.2 ± 0.3) min. Consequently, rePON1 lactonase activity was corrected for spontaneous hydrolysis of the substrate.

### 2.2. Calculating K_m_ and V_max_ from the Initial Rates

The initial rate of the nonenzymatic reaction was calculated for each substrate concentration as v_0_ = k_NE_ · [S]_0_ and subtracted from the appropriate initial rate of the entire reaction (which comprises the enzymatic and nonenzymatic reactions). Such a calculation increases the precision of the v_0_ values.

The initially assumed [S]_0_ values are imprecise because they account for neither (1) small errors in pipetting and (2) the small delay between the beginning of the reaction and the beginning of the measurement. Hence, we used adjusted [S]_0_ values, hereafter denoted as [S]_0_ *. The adjusted [S]_0_ * that corresponds to the initial rate is the substrate concentration calculated retroactively from the progress curve (see equation in Section 4.4, paragraph 2). This can be achieved when the enzyme-catalyzed reaction is irreversible or when the ratio of reactants and products at equilibrium is known.

As the fitted substrate concentrations slightly differ between different programs, we used the values fitted by Prism using the integrated MM equation. The [S]_0_ * values were then used throughout our data analysis due to their accuracy, except for the calculations described in the rightmost two columns of Table 1. The differences between the initially assumed and adjusted substrate concentrations ranged from 0.38% (90.34 vs. 90 μM) to 58% (12.47 vs. 30 μM) (Table 2).

First, it was necessary to assess whether the distribution of initial rates was normal (using the Kolmogorov–Smirnov test). The value of the K-S test statistic (D) was 0.095, and the *p* value was 0.924, indicating that the data did not differ significantly from normally distributed data.

**Figure 1:**
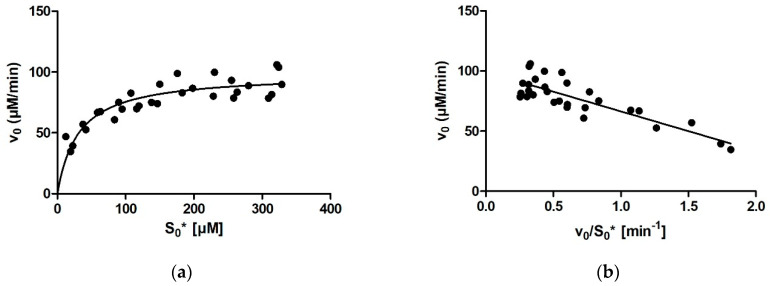

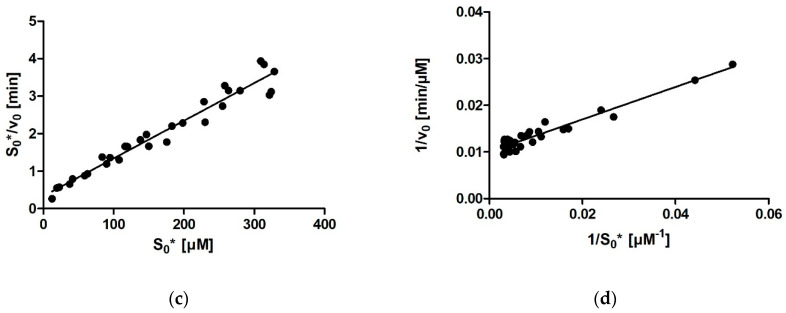
Determination of *K_m_* of rePON1 from the initial rates. (**a**) The Michaelis–Menten diagram: *K_m_* = 31 ± 6 µM. (**b**) The Eadie–Hofstee diagram: *K_m_* = 33 ± 4 µM, R = 0.84, R^2^ = 0.71. (**c**) The Woolf–Hanes diagram: *K_m_* = 34 ± 8 µM, R = 0.98, R^2^ = 0.95. (**d**) The Lineweaver–Burk diagram: *K_m_* = 35 ± 2 µM, R = 0.96, R^2^ = 0.93. The [S]_0_* are the initial substrate concentrations calculated from the progress curves.

No outlier points were present when fitting the initial rates with the MM equation: v_0_ = *V_max_* · [S]/(*K_m_* + [S]). The best-fit values for *K_m_* and *V_max_* were 31 ± 6 µM (95% CI: 31 ± 11 µM) and 99 ± 4 µM/min (95% CI: 99 ± 8 µM/min), respectively (Figure 1a).

With the Eadie–Hofstee method, one outlier point needed to be removed. For 29 points, the calculated *K_m_* and *V_max_* values were 33 ± 4 µM (95% CI: 33 ± 8 µM) and 99 ± 3 µM/min (95% CI: 99 ± 6 µM/min), respectively. The correlation coefficient and R-squared were R = 0.84 and R^2^ = 0.71, respectively (Figure 1b).

With the Woolf–Hanes method, no outlier points needed to be removed. The calculated *K_m_* and *V_max_* values were 34 ± 8 µM (95% CI: 34 ± 16 µM) and 100 ± 1 µM/min (95% CI: 100 ± 1 µM/min), respectively. The correlation coefficient and R-squared were R = 0.98 and R^2^ = 0.95, respectively (Figure 1c).

With the Lineweaver–Burk method, the same point that acted as an outlier in the Woolf–Hanes diagram had to be removed again. From the remaining 29 points, the best-fit values for *K_m_* and *V_max_* were 35 ± 2 µM (95% CI: 35 ± 5 µM) and 100 ± 3 µM/min (95% CI: 100 ± 6 µM/min), respectively. The correlation coefficient and R-squared were R = 0.96 and R^2^ = 0.93, respectively (Figure 1d).

The effect of calculating *K_m_* and *V_max_* without accounting for the nonenzymatic reaction and without adjusting the concentration of S_0_ was also tested. All the *K_m_* and *V_max_* values are shown in Table 1.

### 2.3. Calculating Kinetic Parameters with the Integrated MM Equation in Prism: The Entire Curve

The normality of the distribution of the output *K_m_* values was assessed with the Kolmogorov–Smirnov test. The value of the K-S test statistic (D) was 0.103, and the *p* value was 0.879, indicating that the data did not differ significantly from normally distributed data.

**Figure 2:**
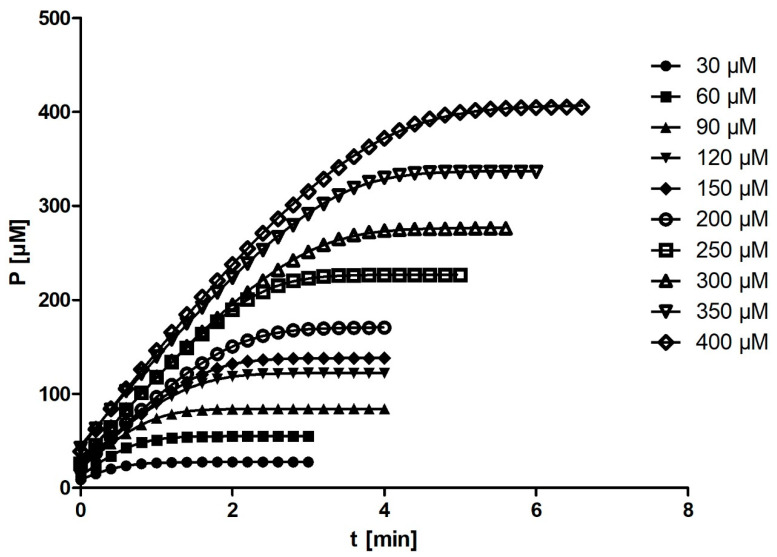
The fit of kinetic progress curve data at different substrate concentrations. Symbols represent absorbance readings converted into concentrations at the given reaction time. For clarity, only one progress curve per experiment and only 1/10 of the data points per progress curve are shown. Smooth lines represent least-square model curves generated by the fit of Equation (4) with the parameter values obtained by the modified model shown in Table 2 (Prism, entire curve). The concentrations shown on the right are the initially assumed ones; the actual (adjusted) concentrations in the cuvettes were calculated from the progress curves and used in all subsequent calculations. The 3rd parallel run is shown. The curves do not originate from the point (0,0); hence, the curves’ plateaus are higher than the S_0_* values displayed in Table 2.

The fit of kinetic progress curve data at different substrate concentrations with the integrated MM equation in Prism (the entire curve) is shown in Figure 2. The kinetic parameters *K_m_* and *V_max_* obtained from the fit depended on the substrate concentration (Table 2, Prism, entire curve). The average of all 30 measurements of *K_m_* for rePON1 was 36 ± 11 µM (95% CI: 36 ± 4 µM); however, these average values are unreliable because the output *K_m_* is dependent on substrate concentration. The average of all 30 *V_max_* values was 109 ± 15 µM/min (95% CI: 109 ± 6 µM/min).

### 2.4. Calculating Kinetic Parameters with Dynafit

The progress curves were analyzed with Dynafit by a numerical integration approach, which is an alternative method for time course data analysis. With Dynafit, the initial values of the microscopic parameters k_1_, k_−1_, and k_2_ must be inserted by the user after the program models the values of these parameters, and the user must calculate *K_m_* from the results. It is also possible for Dynafit to model *K_m_* and *V_max_* directly; however, modeling microscopic parameters allows for the nonenzymatic constant (k_NE_ = 0.084 min^−1^) to be included in the model, which is the main benefit of Dynafit.

The normality of the distribution of output *K_m_* values was assessed with the Kolmogorov–Smirnov test. The value of the K-S test statistic (D) was 0.129, and the *p* value was 0.656, indicating that the data did not differ significantly from normally distributed data. The kinetic parameters *K_m_* and *V_max_* obtained from the fit did not depend on the substrate concentration (Table 2, Dynafit). Thus, the average of the 30 *K_m_* values was calculated from the progress curves and was 27 ± 4 µM (95% CI: 27 ± 1.4 µM). The average of all 30 *V_max_* values was 91 ± 12 µM/min (95% CI: 91 ± 4 µM/min).

### 2.5. Calculating Kinetic Parameters with iFIT

**Figure 3:**
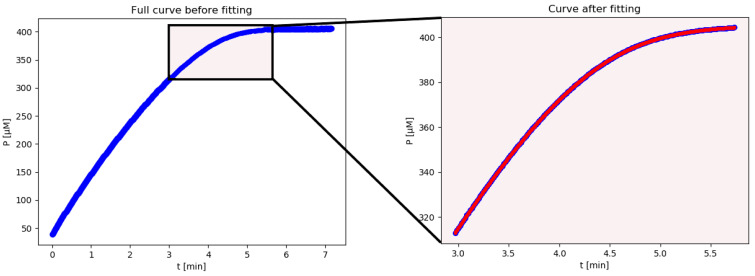
The fit of kinetic progress curve data by iFIT. The entire curve is displayed on the (**left**), whereas only points from the area of highest curvature were included in the fit (**right**). The blue line represents absorbance readings converted into concentration at the given reaction time. Only one progress curve is shown, for clarity. The red line represents the least-square model curve generated by fitting with Equation (4) after the iteration process, with the parameter values obtained by the modified model shown in Table 2 (iFIT). The progress curve is the 400 µM curve from the 3rd parallel run shown in Figure 2 and Table 2.

iFIT uses only a small number of points from a progress curve (see Figure 3 for an illustration of its point selection). Equation (5) indicates that the *t*_Q_ selected for fitting will be roughly proportional to *K_m_*/*V_max_*. Since our progress curves with DHC as a substrate had a low *K_m_* compared to *V_max_* (Table 2), only a small number of points from the progress curve were ultimately analyzed by iFIT. In cases where a progress curve began to be measured immediately and reached its plateau, iFIT will generally trim points both from the left and the right side.

The kinetic parameters *K_m_* and *V_max_* obtained from the fit do not depend on the substrate concentration (Table 2, iFIT). Thus, the average of the 29 *K_m_* values was calculated and was 27 ± 5 µM (95% CI: 27 ± 2 µM). The average of all 30 *V_max_* values was 96 ± 11 µM/min (95% CI: 96 ± 4 µM/min). The normality of the distribution of output *K_m_* values was assessed with the Kolmogorov–Smirnov test. The value of the K-S test statistic (D) was 0.114, and the *p* value was 0.807, indicating that the data did not differ significantly from normally distributed data.

### 2.6. The Relationship between Substrate Concentration and Calculated K_m_

**Figure 4:**
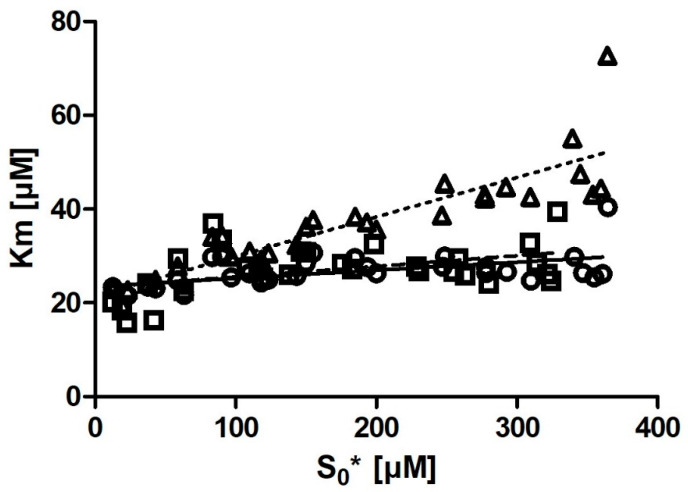
The relationship between adjusted substrate concentration ([S]_0_*) and *K_m_* for Dynafit (circles), iFIT (squares), and Prism (the entire curve) (triangles) for all 30 measurements. Lines represent least-square model curves generated by a linear fit. The correlation coefficients and R-squared of the best-fit lines are R = 0.443 and R^2^ = 0.196 (iFIT), R = 0.525 and R^2^ = 0.276 (Dynafit), and R = 0.887 and R^2^ = 0.787 (Prism: the entire curve), respectively. The concentrations displayed were calculated retrospectively from the progress curves (Table 2).

A very notable characteristic of the output *K_m_* values determined from the progress curves (Table 2) was the positive correlation between *K_m_* and the adjusted substrate concentration ([S]_0_*). The relationship between [S]_0_* and *K_m_* for all three programs is shown in Figure 4. The relationship was linear and varied among the three programs used for fitting. With Dynafit and iFIT, the correlation was weak (or no correlation was seen), and *K_m_* hardly increased with increases in [S]_0_*. When straight lines were fitted onto the relationship between *K_m_* and [S]_0_*, they resulted in the following relationships: *K_m_* = 0.0227 · [S]_0_* + 23.35 µM, with R = 0.443 and R^2^ = 0.196 (for iFIT) and *K_m_* = 0.0173 · [S]_0_* + 23.51 µM, with R = 0.525 and R^2^ = 0.276 (for Dynafit). With Prism (the entire curve), the correlation was strong: *K_m_* = 0.0842 · [S]_0_* + 21.48 µM, with R = 0.887 and R^2^ = 0.787 (Figure 4).

### 2.7. The Relationship between Different Programs for Calculating K_m_ from Progress Curves

**Figure 5:**
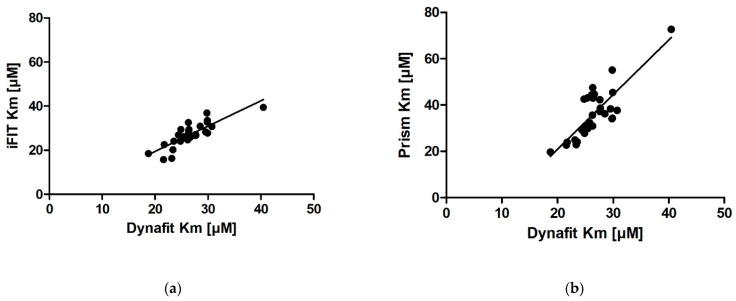

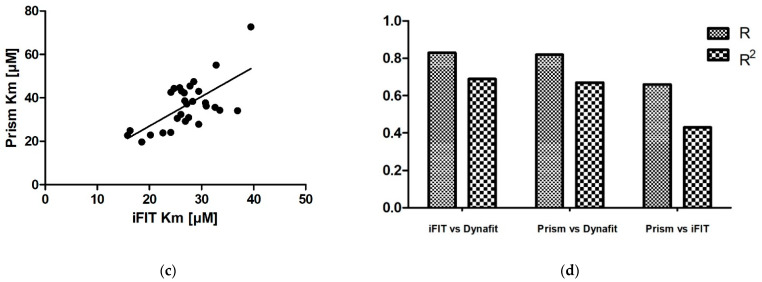
The relationship between output *K_m_* values for the same progress curves analyzed by three different programs: (**a**) iFIT vs. Dynafit, (**b**) Prism (the entire curve) vs. Dynafit, and (**c**) Prism (the entire curve) vs. iFIT. On each graph, all *K_m_* calculations are shown. The linear fits of the relationships are as follows: (**a**) (iFIT *K_m_*) = 1.15 · (Dynafit *K_m_*) − 3.47 µM; (**b**) (Prism (the entire curve) *K_m_*) = 2.36 · (Dynafit *K_m_*) − 26.26 µM; and (**c**) (Prism (the entire curve) *K_m_*) = 1.36 · (iFIT *K_m_*) − 0.17 µM. The R and R^2^ values for all three comparisons are shown in (**d**).

In addition to assessing the relationship between *K_m_* and [S]_0_*, the correlations among the results of the three tools for progress curve analysis were also analyzed (Figure 5). The iFIT–Dynafit and Prism (the entire curve)–Dynafit correlations were strong (R = 0.83 versus R = 0.82 and R^2^ = 0.69 versus R^2^ = 0.67, respectively), while the Prism (the entire curve)–iFIT correlation was moderate (R = 0.66 and R^2^ = 0.43). The *K_m_* values for iFIT were on average slightly higher than those for Dynafit (the slope of the line was 1.15). The *K_m_* values for Prism (the entire curve) were higher than those for both Dynafit and iFIT (the slopes of the lines were 2.36 and 1.36, respectively).

## 3. Discussion

The three main enzymatic activities of PON1 are aryldialkylphosphatase (more commonly known as paraoxonase), arylesterase, and lactonase activity [17]. Most artificial substrates for PON1 fall into one of these three categories. Although lactonase activity has been proposed as the “native” evolutionary activity of human PON1, most experimental studies, especially in medical contexts, continue to be performed on paraoxonase and/or arylesterase substrates [16]. Using different substrate types when comparing groups of test subjects for PON1 enzymatic activity in clinical studies can lead to contradictory results [18,19]. This emphasizes the need to use more than one substrate type when investigating PON1 in a clinical context. This in turn also means that lactonase activity should be more frequently investigated in clinical studies than is presently the case.

The most-used lactone substrate for investigating PON1 is DHC [19]. Despite its relative ubiquity in medical studies, DHC has only been kinetically characterized as a substrate of PON1 in a handful of published articles. Most notably, the *K_m_* value for the reaction between PON1 and DHC has seldom been reported in studies so far: it has been reported, e.g., for human serum PON1 [15,20], for rat serum PON1 [21], and for rePON1 [16]. Certain specific challenges that DHC presents as a substrate, e.g., its rate constant of nonenzymatic hydrolysis (*t*_1/2_ = 8.25 min), have hardly been mentioned in the literature at all. We therefore wish to contribute to a more thorough understanding of the kinetic parameters of PON1-induced DHC breakdown, which should be useful to all future researchers investigating the lactonase activity of PON1 in experimental as well as clinical contexts.

The only *K_m_* value that has been reported in the published literature for DHC and rePON1 G2E6 is 129 ± 8 µM [16]. There is a large discrepancy between this value and our results (*K_m_* was 27, 27, and 31 µM for Dynafit, iFIT, and the MM diagram, respectively; the Prism (the entire curve) average is not included because of the dependence of the output *K_m_* on substrate concentration). Khersonsky and Tawfik [16] did not account for the nonenzymatic hydrolysis of DHC in water, which we have shown to be substantial, and used a relatively high ionic strength for their buffer supplemented with the detergent tergitol. In PON1, we observed that high ionic strength produces higher *K_m_* values when working with DHC. Additionally, not accounting for nonenzymatic hydrolysis produces higher apparent *K_m_* values when calculating *K_m_* from initial rates.

Indeed, in the present data analysis, using the initially assumed S_0_ values instead of the adjusted ones and not subtracting the nonenzymatic reaction from the initial rates resulted in *K_m_* values more than twice as high as those acquired when S_0_ and v_0_ were properly adjusted (Table 1). The *K_m_* value calculated without accounting for the nonenzymatic reaction and without adjusting the concentration of S_0_ was 74 ± 11 μM. This value is closer to the reported value from the literature [16]. Unfortunately, this means that we have no consensus on a specific *K_m_* value against which to compare our results.

The adjusted S_0_* values are those calculated retroactively from the progress curves. They are usually smaller than the initially assumed concentrations because they do not include the amount of substrate that was consumed before the start of the measurement; this is especially relevant for very small substrate concentrations. Most researchers working with initial rates do not record the entire progress curve and thus never know how far off their substrate concentrations might have been from the initially assumed concentrations; this is another advantage of working with entire progress curves, regardless of which analysis we perform afterwards. Nevertheless, once we record the entire progress curve, it makes more sense to directly extract the kinetic parameters from the progress curve rather than use the initial rates approach.

Comparing the different approaches for determining *K_m_* that we used (four based on the MM diagram and two based on progress curves; the approach of fitting the entire curve in Prism is not included here for the abovementioned reasons) reveals that the average *K_m_* value ranges between 27 µM for Dynafit and 35 µM for the Lineweaver–Burk method. The results can be grouped into two clusters, with very similar average results acquired with Dynafit and iFIT (27 µM in both cases) and by the four methods based on the MM diagram (31–35 µM). If we consider all these approaches as equally valid, we can conclude that the *K_m_* of rePON1 for DHC is approximately 30 µM. The clusters are less pronounced for the *V_max_* values. The average *V_max_* values are 99–100 μM/min for all initial rate approaches, 96 μM/min for iFIT, and 91 μM/min for Dynafit.

It is apparent from our results that not all approaches for determining *K_m_* can be considered equally reliable. The integrated MM equation in GraphPad Prism (hereafter referred to as only “Prism” in the Discussion) is particularly problematic because the increasing substrate concentration has a major effect on the *K_m_* output value. The major reason for this is most likely the nonenzymatic hydrolysis of the substrate, a first-order reaction. Hence, as we increase substrate concentration, nonenzymatic hydrolysis will play a proportionally increasing role in the total reaction and will have the greatest impact at the beginning of the progress curve. When fitting the model function to the entire progress curve, the consequence will be that the model function will no longer fit well to the area of maximum curvature (see Figure A1 in Appendix A). DHC is unusual due to its rate of nonenzymatic hydrolysis; however, many other substrates also decompose spontaneously in the absence of an enzyme. This can be especially problematic if researchers are not aware of the substrate’s properties.

In the case of rePON1 and DHC, iFIT and Dynafit present two different approaches that minimize the effect of nonenzymatic hydrolysis on the final value of *K_m_*. Intuitively, we might expect Prism and iFIT to produce similar results (since they both use the same integrated MM equation that does not directly account for side reactions) and Dynafit to produce different results (since it uses a system of differential equations and accounts for nonenzymatic hydrolysis). When calculating *K_m_* and *V_max_* in Dynafit, the user must calculate the three microscopic parameters, k_1_, k_−1_, and k_2_, separately and then calculate *K_m_* from them according to Equation (2).

We obtained very similar results with iFIT and Dynafit, and these results differ from those obtained with Prism. The iFIT-Dynafit correlation was strong (R = 0.83). This strongly suggests that both iFIT and Dynafit successfully subtract the contribution of the nonenzymatic reaction from the progress curve: Dynafit by direct modeling and iFIT by considering only data at timescales insensitive to the nonenzymatic reaction, i.e., using only a small number of points around the area of maximum curvature. There, the relative contribution of the spontaneous reaction is negligible, because the latter is pseudo-first-order.

Of the other two correlations, the Prism–Dynafit correlation was equally strong (R = 0.82), while the Prism–iFIT correlation was moderate (R = 0.66). This is counterintuitive, as Prism and iFIT use the same algorithm (the integrated MM equation). This implies that the selection of points is more important than the type of algorithm used for fitting. Prism does not account for the contribution of the nonenzymatic reaction, whereas iFIT does. Dynafit and Prism both used the same points; this would explain why the correlation between them was strong.

In addition to reducing the influence of nonenzymatic reactions on output kinetic parameters, our motivation behind developing iFIT was to establish a method for determining *K_m_* that is more precise than working with entire progress curves. The precision of iFIT, i.e., the small dispersal of output *K_m_* values around the mean, is evident, as the standard deviations of the calculated results were much smaller than those for the integrated MM equation in Prism (Table 2). Furthermore, the increase in *K_m_* with substrate concentration was also much smaller. Even more notably, we observed that if we removed the six output *K_m_* values obtained from the progress curves with the lowest substrate concentration (Figure 4), the correlation between substrate concentration and *K_m_* disappeared entirely.

Another issue of interest to experimenters is the optimal concentration of the substrate for progress curve measurements. Several articles have discussed, mostly based on data produced in silico, the impact of substrate concentration on the magnitude of error of estimating *K_m_* values [2,8,10]. In some cases, this also led to recommendations regarding the ideal concentration range of the substrate; however, this range differs between different fitting methods. For example, Duggleby and Clarke [2] suggested a [S]_0_ value around 2.5 · *K_m_* to minimize error, while they claimed that their acquired *K_m_* values were independent of substrate concentration. For the method we used in iFIT, the recommendation (by Stroberg and Schnell) that [S]_0_ should be in the order of magnitude of *K_m_* is especially pertinent.

For iFIT, substrate concentration is expected to be less relevant. This is because the script only works with a small area of the progress curve that corresponds to a substrate concentration in the order of magnitude of *K_m_* (which depends on the *V_max_* value from which the area of maximum curvature is also calculated). If possible, it is advised to not go below this [S]_0_, as this would deprive iFIT of some of the points it uses for analysis.

In the case of DHC, because of the extinction coefficient ε = 1310 M^−1^ cm^−1^, it is difficult to measure reactions with <15 µM DHC (as the noise obscures the signal) or >800 µM DHC (as the upper limits of most spectrometers’ sensitivities are reached). We observed from our data that the *K_m_* values calculated from progress curves with a low substrate concentration were slight outliers in iFIT. We also observed that iFIT can encounter issues with progress curves measured at a very low substrate concentration. Consequently, the optimum substrate concentration for DHC is between 2 and 4× *K_m_*. For other enzyme–substrate reactions, we would like to amend Stroberg and Schnell’s recommendation and suggest using at least 2× *K_m_* as the initial substrate concentration in order to seize the full potential of the data-point-reduction approach that we present in the current article.

It is worthwhile to be aware of the circumstances where iFIT would not be superior to working with whole curves, for the reason that the deviations from an MM mechanism are most pronounced closer to the plateau of the progress curve, i.e., around the area of maximum curvature. This could be the case when we have enzyme inhibition by product. Unfortunately, the solution in such cases would not be to produce an alternative program, which would exclude points in the area of maximum curvature, since those points would continue to encode the greatest amount of information about *K_m_*.

To test the performance of iFIT on potentially troublesome curves for reactions which include inhibition by product, we compared the different programs on two additional sets of progress curves for the enzymes: (1) penicillin amidase, cleaving the artificial substrate NIPAB, with data that had already been published by Zavrel et al. [10] and reanalyzed by Goličnik [22], and (2) butyrylcholinesterase, cleaving the artificial substrate butyryltiocholine, with our own set of measurements, based on methods used by Stojan [23]. Both of these results are presented fully in the Supplementary Material. In the case of penicillin amidase, it is clear that iFIT is comparable to fitting the entire progress curves, despite not directly accounting for product inhibition. Additionally, in the case of butyrylcholinesterase, iFIT did not perform any worse in determining the accurate value of *K_m_* than the integrated MM equation in Prism. The implication of both of these sets of results is that users should not be afraid to replace the whole-curve approach with iFIT.

At the same time, it is worth pointing out that researchers should not immediately assume a progress curve follows classic Michaelis–Menten kinetics. If the enzyme exhibits features such as cooperativity, which means that one cannot define the reaction using *K_m_* and *V_max_*, then of course iFIT cannot be used for progress curve analysis. Similar considerations hold for reaction mixtures with several enzymes, none of which contribute predominantly to the overall reaction. Before using iFIT, researchers should check whether MM kinetics is a reasonable approximation for the behavior of their enzyme.

## 4. Materials and Methods

### 4.1. Chemicals

The substrate DHC, methanol, calcium chloride, and all other chemicals for the expression and purification of rePON1 were purchased from Sigma Aldrich (St. Louis, MO, USA). TRIS was from Carl Roth GmbH (Karlsruhe, Germany). Nickel–nitrilotriacetic acid was from Qiagen (Hilden, Germany). Plasmid containing the G2E6 variant of the rePON1 gene was kindly provided by Prof. Daniel S. Tawfik from the Weizmann Institute (Rehovot, Israel).

### 4.2. Recombinant PON1 Expression and Purification

The G2E6 variant of rePON1 was used as a source of enzyme. RePON1 protein was expressed and purified in the *Escherichia coli* bacterial system according to the procedures reported previously [14] with minor modifications [24].

A single colony obtained after transformation with plasmid pET32b (+)-rePON1 into Origami B (DE3)pLysS cells was used to inoculate 10 mL of LB medium with 100 µg/mL ampicillin, 25 µg/mL chloramphenicol, and 1 mM CaCl_2_. The culture was grown at 37 °C for 17 h. Then, 500 mL of LB medium containing 100 µg/mL ampicillin, 25 µg/mL chloramphenicol, and 1 mM CaCl_2_ was inoculated with 5 mL of overnight culture and grown at 37 °C to an OD600 of 0.7. The expression of the rePON1 variant was induced by adding 1 mM isopropyl β-D-1-thiogalactopyranoside, and the culture was grown at 25 °C for 17 h. The cells were harvested by centrifugation at 6000× *g* for 15 min, and the pellet was stored overnight at −20 °C. The cells were resuspended in 30 mL of lysis buffer (50 mM Tris, pH = 8.0, 1 mM CaCl_2_, and 0.1 mM dithiothreitol supplemented with 1 µM pepstatin A, 1 mM phenylmethylsulfonyl fluoride, and 0.03% n-dodecyl-β-D-maltopyranoside (C12-maltoside)) and lysed by sonification. The lysate was centrifuged at 10,000× *g* for 10 min, and the supernatant was stirred for 1 h at 4 °C. After centrifugation at 20,000× *g* for 20 min, the soluble fraction was treated with ammonium sulphate (55%, *w*/*v*, at 0 °C). The precipitate was centrifuged at 10,000× *g* for 15 min, resuspended, and dialyzed twice against lysis buffer supplemented with 0.01% C12-maltoside. After dialysis, the protein was added to nitrilotriacetic acid resin, and the mixture was gently shaken overnight at 4 °C. The resin was first washed with lysis buffer with 0.03% C12-maltoside and then with 10 and 20 mM imidazole in lysis buffer with 0.03% C12-maltoside. It was finally eluted with 150 mM imidazole in lysis buffer with 0.03% C12-maltoside. Fractions with the highest rePON1 activity were pooled, dialyzed, and further purified by ion-exchange chromatography. The protein was applied to a 5 mL HighTrap Q HP column (GE Healthcare, City, Marlborough, MA, USA) with a linear gradient from 26% to 33% of buffer B (20 mM Tris, pH = 8.0, 1 mM CaCl_2_, 0.1 mM dithiothreitol, 0.03% C12-maltoside, 1 M NaCl) in buffer A (buffer B without 1 M NaCl). Fractions with the highest rePON1 activity were analyzed on an 11% SDS-PAGE gel, pooled, dialyzed against buffer A, and concentrated. Finally, sodium azide (0.02%) was added, and the protein was stored at −70 °C.

The purity of rePON1 (95%) was finally assessed by SDS-PAGE, and the concentration of rePON1 was determined using the Bradford assay. A stock solution of 2 µM (0.13 mg/mL) rePON1 was used for all measurements. The final enzyme concentration in assay was 0.01 µM.

### 4.3. Measurements of the Lactonase Activity of RePON1

DHC was used as a substrate for the enzymatic reaction. The stock solution was prepared by diluting the substrate in methanol to a final concentration of 2.5 mM. From this stock solution, 10 different final concentrations of substrate (30, 60, 90, 120, 150, 200, 250, 300, 350, and 400 µM) were prepared in methanol (1% in all the reaction mixtures).

The enzymatic reaction was performed in a 1 cm cuvette with a total volume of 2 mL. The hydrolysis of DHC was measured in 50 mM Tris–HCl buffer (pH = 8.0) containing 1 mM CaCl_2_ at 25 °C using a Genesys 10S spectrophotometer (Thermo Fisher Scientific, Waltham, MA, USA). A total of 10 µL of rePON1 was added to the buffer, corresponding to a final concentration of 0.01 µM. After the addition of 20 µL of DHC, the increase in absorbance was measured at 270 nm (ε = 1310 M^−1^ cm^−1^). Absorbance measurements were obtained every second. The entire progress curve was recorded, and the recording was stopped when the curve plateaued for at least 1 min. Each progress curve was recorded in three independent experiments, resulting in a total of 30 measurements. The rates (v_0_) of spontaneous hydrolysis for all 10 substrate concentrations were also measured. Consequently, rePON1 activity was corrected for spontaneous hydrolysis of each substrate.

### 4.4. Determination of K_m_ of RePON1

Different approaches were used and compared to extract the values of the kinetic parameters *K_m_* and *V_max_* from the progress curves. Three different programs for progress curve analysis as well as the classical approach of measuring initial rates were used. When working with initial rates, three different versions of data linearization were used; the MM equation was also fitted directly onto the initial rate data points.

The three programs for progress curve analysis (Dynafit, Prism, and iFIT) also calculated the initial substrate concentration from the extinction coefficient of the reaction’s product and the difference in absorbance at 270 nm between the beginning and end of the measurement: −ΔS [μM] = ΔP [μM] = ΔAbs [arb. units]/(ε · l). The assumed substrate concentrations are provided in Section 4.3. However, thereafter, any reported substrate concentrations were calculated directly from each individual progress curve; we refer to them as “adjusted” substrate concentrations.

#### 4.4.1. Initial Rate Measurements

The initial rate (v_0_) values can differ based on how many points they are calculated from [25]. To acquire the most precise possible estimate, v_0_ was not calculated by fitting a straight line directly onto the beginning of the curve, as this would only give an intermediate value for the reaction rate during the early part of the reaction. Instead, we calculated v_0_ at *t* = 0 from the first derivative of the progress curve, as described by Hasinoff [26].

For calculating enzymatic parameters from v_0_, the following were compared: (1) the MM analysis provided in the GraphPad Prism package, (2) the Lineweaver–Burk method, (3) the Eadie–Hofstee method, and (4) the Hanes–Woolf method. The necessary parameters (*K_m_*, *V_max_*, and the quality-of-fit estimates) were calculated in Prism in all four cases.

#### 4.4.2. Numerical Integration of Differential Equations in Dynafit

**Figure 6:**
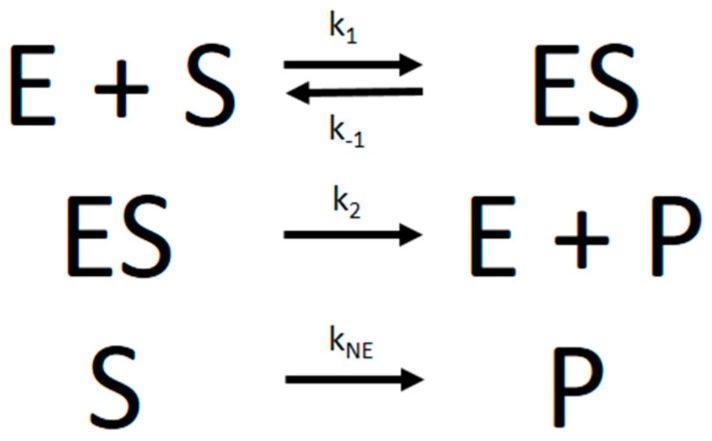
The Michaelis–Menten reaction scheme, with the nonenzymatic decay of substrate added in the bottom line. The formation of the enzyme–substrate complex is the “slow” step of the overall reaction, whereas the formation of product is the “fast” step. If k_−1_ = 0, the reaction proceeds according to the Van Slyke–Cullen mechanism.

The progress curve experimental data were analyzed with the nonlinear regression fitting program Dynafit [5] using numerical integration of differential equations that describe the simple MM reaction model in Figure 6:

From the microscopic rate constants, *K_m_* can then be calculated according to Equation (2):

(2) $$K_{m}=\frac{k_{2}+k_{-1}}{k_{1}}$$

Besides considering the basic kinetic parameters that determine *K_m_* and *V_max_*, i.e., the rate constants *k*_1_, *k*_−1_, or *k*_2_, Dynafit is the only program that can also include additional kinetic parameters in the model. Most importantly, it can include a kinetic constant for nonenzymatic substrate hydrolysis (which we denoted as k_NE_), an important factor in several PON1-mediated enzymatic reactions.

#### 4.4.3. An Integrated MM Rate Equation in Prism: The Entire Curve

The second program that was used to analyze the progress curves was GraphPad Prism (hereon: Prism), which allows user-generated equations for modeling data. The equation implemented in Prism was based on the integrated MM equation that had been originally described by Stroberg and Mendoza ([3]; see Equation (3)).

(3) $${\left[P\right]}_{t}={\left[S\right]}_{0}-K_{m}\cdot W_{0}\left(\frac{{\left[S\right]}_{0}}{K_{m}}\cdot \exp\left(\frac{{\left[S\right]}_{0}-V_{max}\cdot t}{K_{m}}\right)\right)$$

For analysis in Prism, the algebraic solution of this equation was used (Equation (4)), as described by Goličnik [4]:

(4) $${\left[P\right]}_{t}={\left[S\right]}_{0}-K_{m}\cdot (1.45869\cdot \ln\left(1.2\cdot \frac{x}{\ln\left(2.4\cdot \frac{x}{\ln\left(1+2.4x\right)}\right)}\right)-0.45869\cdot \ln\left(2\frac{x}{\ln\left(1+2x\right)}\right))$$

The program uses Equation (4) to fit the entire progress curve and directly outputs the parameters *K_m_* and *V_max_* as well as the errors for these parameters. Unfortunately, there is no automatic (scripted) way within Prism to exclude experimental points from the fit. Unnecessary points are especially problematic for curves that continue for a long time after plateauing. Consequently, imperfect fits are made at the expense of the area of maximum curvature (Figure A1 in Appendix A), which contains the most information about *K_m_* and *V_max_*.

#### 4.4.4. An Integrated MM Rate Equation in iFIT: The Area of Highest Curvature

An equation for calculating the portion of the time course (*t_Q_*) over which the progress curve exhibits the maximal curvature based on *K_m_* and *V_max_* values was presented by Stroberg and Schnell (Equation (5)) [7]:

(5) $$t_{Q}=\frac{27K_{m}\cdot {\left[S\right]}_{0}}{4V_{max}\left(K_{m}+{\left[S\right]}_{0}\right)}$$

Based on their theoretical work, we have developed the computer script iFIT to alleviate the problem mentioned in Section 4.4.3 and determine *K_m_* and *V_max_* from the high-curvature regions of progress curves. iFIT uses a script in Python to remove data points on both sides of the maximum-curvature region and fits the rest of the progress curve with Equation (4).

iFIT is an iterative method that first calculates *K_m_* and *V_max_* from the entire available progress curve using the integrated Michaelis–Menten equation described above. From these, it then calculates the width of the area of maximum curvature, denoted as *t_Q_*, and removes all points from the progress curve, which are more than *t_Q_*/2 away from the point of inflection. From the remaining points, it then calculates *K_m_* and *V_max_* with the integrated MM equation again, uses these to calculate *t_Q_*, and so on, until the calculated *t_Q_* converges to the same value, i.e., the selected experimental time–concentration points are the same in two successive iterations. If this does not happen within 100 iterations, the program reports all 100 steps at the end of the calculation. The iterations may not converge when there is excessive noise in the experimental data. In such cases, the program might oscillate between two or more *t_Q_* values, or *t_Q_* might drift outside the actual area of experimental data points, in which case there will not be an output result.

The advantage of this method is that the exact time when we start and stop measuring the reaction is less relevant since any superfluous points will be removed by the script. In conclusion, the advantage of iFIT is that it can iteratively adjust the region of data that will be used to fit the model equation.

### 4.5. Statistics

*K_m_* and *V_max_* are expressed as mean ± standard deviation. Additionally, the correlation coefficients, the 95% confidence intervals, and R-squared goodness-of-fit measures for linear regression models are also shown for each group of data. For all the groups of output values, the normality of their distribution was calculated with the Kolmogorov–Smirnov test. Differences were considered statistically significant at *p* < 0.05 (GraphPad Prism).

## 5. Conclusions

The present study only investigated rePON1, which is commonly used to investigate the catalytic function of PON1 [14,20,27]. There is little reason to expect that the methods for fitting that we presented in this study would have performed very differently if we had used a naturally occurring PON1 variant. However, one aspect that cannot be studied with rePON1 is the interindividual variability of enzyme activity and thus the ability of different methods to measure this variability.

Measuring kinetic parameters, particularly *K_m_*, continues to be highly underutilized in studies that measure human PON1 activity in various medical conditions. This is particularly the case with lactonase activity; not a single study presented in Petrič et al. [19] measured *K_m_* in human patients. Furthermore, it has been suggested that *K_m_* should be more routinely included by researchers when comparing interindividual enzymatic activity to detect differences that might not be noticed in a simple comparison of a specific activity [28]. By providing a framework for measuring the *K_m_* of PON1 for DHC, we hope that we have facilitated future studies of such kinds on PON1.

The most important contribution of our study is the practical application of Stroberg and Schnell’s equation on experimental enzymatic data, which has been made possible with a Python script iFIT that quickens an otherwise very cumbersome iterative process. We thus very strongly recommend that researchers who wish to determine *K_m_* values from progress curves consider such a method, which selects points from the curve’s maximum curvature area, as this increases the precision of their fitted *K_m_* values.

## Figures and Tables

**Table 1:** The *K_m_* and *V_max_* values for all four approaches for determining the kinetic parameters from the initial rates. The left side of the table shows the *K_m_* and *V_max_* values that were acquired by calculating substrate concentration from the progress curve (i.e., [S]_0_*) and by accounting for the nonenzymatic hydrolysis of substrate. The right side shows the *K_m_* and *V_max_* values when these two factors were not accounted for. All values are shown with standard deviations.

Method	[S]_0_ * Adjusted, Nonenzymatic Reaction Accounted for		[S]_0_ Not Adjusted, Nonenzymatic Reaction Not Accounted for	
	*K_m_* (µM)	*V_max_* (µM/Min)	*K_m_* (µM)	*V_max_* (µM/Min)
MM diagram	31 ± 6	99 ± 4	74 ± 11	138 ± 6
Eadie–Hofstee	33 ± 4	99 ± 3	62 ± 7	130 ± 5
Woolf–Hanes	34 ± 8	100 ± 1	76 ± 10	138.0 ± 0.2
Lineweaver–Burk	35 ± 2	100 ± 3	76 ± 5	137 ± 6

**Table 2:** The values of [S]_0_*, *K_m_*, and *V_max_* for all 30 rePON1 measurements that were calculated by three programs for progress curve analysis: Dynafit, Prism (i.e., the integrated MM equation: the entire curve), and iFIT (i.e., the integrated MM equation: the area of the highest curvature). The [S]_0_ values in the leftmost column are the initially assumed concentrations. The adjusted [S]_0_ values ([S]_0_*) were calculated retroactively from the progress curves. AVG: average; STDEV: standard deviation.

		[S]_0_* (μM)			*K_m_* (μM)			*V_max_* (μM/Min)		
	[S]_0_ (μM)	Dynafit	Prism (Entire Curve)	iFIT	Dynafit	Prism (Entire Curve)	iFIT	Dynafit	Prism (Entire Curve)	iFIT
1st parallel run	30	12.50	12.47	12.49	23.36	22.89	20.20	133.30	133.90	122.20
	60	37.54	37.47	24.81	23.53	24.09	24.08	91.00	96.49	96.28
	90	90.23	90.34	90.10	29.86	34.27	33.50	96.50	109.40	107.21
	120	109.70	109.80	109.48	26.30	30.99	27.52	96.10	109.60	102.38
	150	154.93	154.90	154.64	30.72	37.69	30.75	101.40	118.70	107.24
	200	184.80	184.90	184.17	29.54	38.34	28.27	107.60	127.20	110.39
	250	247.30	246.60	246.01	27.70	38.61	26.78	104.20	127.00	108.06
	300	278.10	277.10	276.54	26.39	43.05	29.45	80.00	105.00	89.03
	350	340.72	339.50	338.87	29.84	55.11	32.78	79.50	110.40	88.87
	400	347.10	345.10	344.23	26.34	47.49	28.51	84.40	113.90	92.67
2nd parallel run	30	23.19	23.18	23.18	21.57	22.73	15.79	81.70	87.55	72.04
	60	58.65	58.69	58.61	24.85	27.82	29.46	83.50	92.89	94.94
	90	83.31	83.36	83.22	29.79	34.09	36.89	82.00	94.00	96.71
	120	123.27	123.30	123.01	24.97	30.59	25.32	82.40	96.41	87.58
	150	143.25	143.20	142.84	25.77	32.37	26.04	83.50	98.83	88.56
	200	193.57	193.30	192.90	27.64	37.18	27.18	89.00	108.30	93.61
	250	277.90	277.30	276.78	27.60	42.33	26.68	96.00	121.40	100.30
	300	309.90	309.40	308.78	24.78	42.54	24.10	92.70	120.20	96.85
	350	360.88	359.60	358.99	26.14	44.36	24.73	104.40	135.10	108.77
	400	354.96	353.80	385.88	25.42	43.10	26.17	108.00	138.10	113.57
3rd parallel run	30	19.19	19.18	19.18	18.71	19.68	18.53	72.10	77.13	74.25
	60	42.93	42.95	42.90	23.13	24.88	16.27	93.30	100.80	85.69
	90	63.25	63.28	63.17	21.72	23.91	22.58	88.10	96.82	94.31
	120	96.75	96.79		25.42	29.83		81.90	94.01	
	150	118.02	118.00	117.78	24.40	29.25	26.90	85.00	98.25	93.52
	200	149.98	150.00	149.69	28.49	36.23	30.89	84.40	101.10	93.59
	250	200.12	199.80	199.40	26.29	35.61	32.59	91.80	111.20	107.15
	300	248.88	248.50	248.02	29.93	45.42	27.77	84.60	108.90	88.29
	350	292.84	292.00	291.46	26.62	44.72	25.81	83.80	110.40	87.69
	400	364.62	364.40	363.11	40.45	72.72	39.44	88.70	125.00	96.45
AVG					26.58	36.40	27.07	91.03	108.93	96.49
STDEV					3.84	11.02	5.38	12.01	14.95	11.15

## Data Availability

We received a series of progress curves that had already been analyzed by Zavrel et al. [10].

## Supplementary Materials

The following are available online, Table S1: The values of *K_m_* for Data set 1 that were calculated by Prism, iFIT and Dynafit for penicillin amidase, Table S2: The values of *K_m_* for Data set 2 that were calculated by Prism, iFIT and Dynafit for rat butyrylcholinesterase.
