# Supplementary material for: The Removal of Time–Concentration Data Points from Progress Curves Improves the Determination of Km: The Example of Paraoxonase 1
Source: Molecules. 2022 Feb 15;27(4):1306. doi: 10.3390/molecules27041306 (PMC8874660; doi:10.3390/molecules27041306)
Supplement: Supplementary file 1 [file molecules-27-01306-s001.zip › molecules-1557175-supplementary.pdf]

Supplementary material

To test the iFIT script on other enzyme-substrate combinations than rePON1-DHC, we performed a series of experimental measurements as well as an analysis of already published data. We were interested in enzymatic reactions with different kinds of side reactions taking place. An especially common problem in enzymatics is the binding of product to enzyme, i.e. product inhibition. Since product inhibition is most pronounced as the reaction approaches its plateau and product concentration is largest, we would expect iFIT to perform less well under such circumstances. Both of the following examples were chosen to test that.

Data set 1: Penicillin amidase

We received a series of progress curves that had already been analyzed by Zavrel et al. [10] and by Goličnik [22]. In the reaction, the enzyme penicillin amidase cleaved the artificial chromogenic substrate NIPAB (2-nitro-5-phenoxyacetamido benzoic acid) into two products, ANB (5-amino-2-nitrobenzoic acid) and PAA (phenylacetic acid); the experimental details are described by Zavrel et al. [10]. The product PAA is known to act as a competitive inhibitor of the enzyme.

The substrate concentration was varied from 75 to 150 μM and the initial concentration of PAA was varied from 0 to 500 μM. The authors of the original article compared 5 different software packages to determine K_m based on the correct reaction model; with each package, they used a global fitting procedure to determine K_m from all curves at once. Each of their progress curves consisted of 48 data points. Both Zavrel and Goličnik acquired K_m values between 30 and 40 μM; in Zavrel, the greatest relative difference in output K_m between two programs was 30%.

We compared three different approaches, all of which fitted the data with a simple Michaelis-Menten model, without any side reactions or product inhibition: Dynafit, the integrated MM equation in Prism, and iFIT. Each curve was fitted separately. Unfortunately, we could not calculate any K_m values from initial velocities, since only three of their progress curves had the same initial product concentration.

For the values of $[S]_0$*, we took the output values calculated by Prism, which are more accurate than the initially presumed $[S]_0$ given by the authors.

Table S1: The values of K_m for Data set 1 that were calculated by Prism, iFIT and Dynafit (using the simple MM model without any side reactions) for penicillin amidase. *$[S]_0$* values were fitted by Prism, whereas* $[P]_0$ values are the ones given by Zavrel et al.

Experiment number	**$[S]_0$* (μM)**	**$[P]_0$ (μM)**	**K_m (Prism) (μM)**	**K_m (iFIT) (μM)**	**K_m (Dynafit - MM model) (μM)**
1	70.96	*0*	60.8	63.2	59.4
2	96.1	*0*	43.4	32.9	26.7
3	147.3	*0*	88.6	82.4	87.8
4	101.5	*50*	60.7	64.2	75.8
5	96.8	*75*	100.6	76.7	100.9
6	108.2	*100*	341	118.1	343

7	99.9	*200*	171.9	143.3	165
8	101.6	*500*	475	233	474

It follows from elementary enzyme kinetics that if $[S]_0$ is held constant, apparent K_m should increase with $[P]_0$,. This is the case with iFIT, but not with the whole-curve approaches, where the output K_m values oscillate considerably. Hence, if we are interested in determining $K_{m(app)}$ from progress curves for reactions that contain product inhibition, it follows that iFIT will produce more sensible results than the whole-curve approach.

Data set 2: Rat butyrylcholinesterase

We also performed a set of measurements ourselves on the often-studied enzyme butyrylcholinesterase (BChE), which is present in vertebrate serum. We tested the enzymatic properties of rat serum, which was taken from a live rat, processed, stored at 4°C and analyzed within one week. The artificial substrate used was butyrylthiocholine (BTCh), which is cleaved by the enzyme into butyrate and thiocholine; Ellmann reagent then reacts with thiocholine to form the products 2-nitrobenzoate-5-mercaptothiocholine and 5-thio-2-nitrobenzoate. The latter is measured spectroscopically at 412 nm. We followed the same protocol used by Stojan [23]. Briefly: the reaction was performed in phosphate buffer, pH = 7, with a final Ellmann reagent concentration of 2 mM. The total volume of each reaction was 600 μM, which included 20 μL of serum; serum and Ellmann reagent were pre-incubated for 15 min, since the reagent may react with the enzyme's side groups.

Substrate concentration varied from 12 to 350 μM. All progress curves were recorded until well after reaching the plateau. In several cases, we added additional highly concentrated substrate to a reaction mixture after the reaction was over, and restarted measuring, to observe the impact of product inhibition on reaction rate. The initial product concentrations, $[P]_0$*, in such cases were taken to be the same as the sum of $[S]_0$* and $[P]_0$* for the previous reaction. Altogether, 14 progress curves were recorded, 8 without initial product presence and 6 with product present at the beginning.

K_m was also calculated from initial velocities, both for $[P]_0$* = 0 and for [P]* = 100 μM; for the latter, we did not take the actual initial velocity of any reaction, but rather the velocity of different reactions at points where total product concentration in the cuvette was 100 μM; $[S]_0$*, in this case, was the remaining substrate concentration in the cuvette. We acquired the following results: K_m = 61 μM for $[P]_0$* = 0; $K_{m(app)}$ = 69.5 μM for [P]* = 100 μM. From these, we then calculated K_i = 750 μM for product inhibition.

We calculated K_m with Prism (integrated MM equation), iFIT, and Dynafit (using a reaction mechanism which included the step E + P <-> EP, and the correct value of $[P]_0$ ($[P]_0$*) when necessary; for K_i, we input the above value of 750 μM). As in the example of penicillin amidase, for $[S]_0$*, and consequently [P]* or $[P]_0$*, we took the values calculated by Prism.

Table S2: The values of K_m for Data set 2 that were calculated by Prism, iFIT and Dynafit (using the correct reaction model with known K_i and correct $[P]_0$ values) for rat butyrylcholinesterase. $[S]_0$ values were fitted by Prism, whereas $[P]_0$* values were calculated by adding together $[S]_0$* and $[P]_0$* for the previous reaction.*

$[S]_0$*(μM(	$[P]_0$* (μM)	K_m (Prism) (μM)	K_m (iFIT) (μM)	K_m (Dynafit, correct model and $[P]_0$*) (μM)
12.85		34.33	21.93	32.22
25.89		32.81	30.81	30.36
79.67		52.6	47.94	44.31
141.8		62.61	63.17	52.14
187.5		60.33	79.35	62.71
356.9		150.8	137.33	88.42
23.29		36.97	33.39	33.88
37.66	23.29	55.02	59.17	47.33
23.58	60.95	50.8	53.93	42.73
23.66	84.53	62.09	N.D	48.05
25.55	108.19	69.55	88.42	54.57
47.79		53.02	57.98	45.76
65.97	47.79	65.94	69.44	52.81
62.13	113.76	90.38	94	66.7

Just like in the first example, and as expected, it is clear that apparent K_m increases with product concentration. Interestingly, this trend exists in Dynafit as well, despite accounting for product inhibition.
